# Direct interaction between the catalytic subunit of Protein Phosphatase 1 and pRb

**DOI:** 10.1186/1475-2867-6-3

**Published:** 2006-02-08

**Authors:** Michele Vietri, Mariarita Bianchi, John W Ludlow, Sibylle Mittnacht, Emma Villa-Moruzzi

**Affiliations:** 1 Department of Experimental Pathology, University of Pisa, 56100 Pisa, Italy; 2 Tengion, Inc., 3929 Westpoint Blvd, Winston-Salem, NC 27103, USA; 3 Centre for Cell and Molecular Biology, Chester Beatty Laboratories, SW3 6JB London, UK

## Abstract

**Background:**

The product of the retinoblastoma-susceptibility gene (pRb) is a substrate for Protein Phosphatase 1 (PP1). At mitotic exit, all three PP1 isoforms, α, γ1 and δ, bind to pRb and dephosphorylate its Ser/Thr sites in a sequential and site-specific way. The pRb-C terminal has been reported to be necessary and sufficient for PP1α binding. The present study investigated whether the three PP1 isoforms from mitotic or asynchronous HeLa cells associate differentially with wild-type and pRb mutants, as well as the holoenzyme composition of the pRb-directed PP1.

**Results:**

The requirement for the entire pRb molecule to achieve optimal PP1-binding was indicated by the fact that full-length pRb displayed the highest affinity for all three PP1 isoforms. Ser/Thr-to-Ala substitution for up to 14 pRb sites did not affect the ability of pRb to bind the PP1 isoforms derived from mitotic or asynchronous HeLa cells, thus suggesting that the phosphate-accepting residues on pRb do not regulate the interaction with PP1. To probe for the presence of PP1 targeting subunits in the pRb-directed PP1 complex, PP1 from mitotic or asynchronous HeLa cells was isolated by affinity chromatography on GST-Rb (either full-length or its deletion mutants Rb-big pocket or Rb-C-terminal). The PP1 was always obtained as free catalytic subunit, displaying all three isoforms, thus suggesting direct interaction between pRb and PP1. The direct association was confirmed by the ability of pRb to pull-down purified PP1 catalytic subunits and by in vitro reconstitution of a complex between PP1 catalytic subunit and the pRb-C-terminal.

**Conclusion:**

The work indicated that the full length of the pRb molecule is required for optimal interaction with the PP1 isoforms and that the association between pRb and PP1 isoforms is direct.

## Background

Type 1 protein phosphatase (PP1), one of the major cellular serine/threonine phosphatases, is abundantly expressed in virtually all cell compartments [1]. PP1 plays a key role in the regulation of cell cycle progression and is also involved in other processes, including gene expression, muscle contraction, glycogen metabolism and neurotransmission [2,3]. The PP1 holoenzyme generally consists of a catalytic subunit bound to a regulatory subunit. Various unrelated regulatory subunits were described, which modulate the catalytic activity and restrict its sub-cellular localization [1,2]. Three PP1 catalytic subunits exist in mammalian cells, α, γ1 and δ (also called β). In spite of being different gene products, these isoforms differ significantly only at their C-termini [4]. Using isoform-specific antibodies developed in this laboratory, these subunits were found to differ in sub-cellular localization, suggesting that they perform different functions [5,6].

One of the physiological substrates of PP1 is the product of the retinoblastoma gene, pRb [7], which controls cell proliferation by regulating the G1-S-phase transition [7,8]. pRb interacts with a variety of cellular proteins dependent upon the phosphorylation state of its 16 Ser/Thr residues. These residues are generally sites of cyclin-dependent kinase (Cdk) phosphorylation [9], which varies as a function of cell cycle phase [10-12]. During early G1, pRb is hypophosphorylated and active as growth suppressor. Hypo-phosphorylated pRb complexes with and sequesters the E2F family of transcription factors, thereby preventing the transcription of genes required for S-phase entry [13]. In middle to late G1, phosphorylation of pRb by Cdks results in the release and activation of E2F and other pRb-bound transcription factors, which than activate the transcription of S-phase genes [14]. During the M-to-G1 transition, pRb is progressively dephosphorylated by PP1, returning to its growth-suppressive hypophosphorylated state [15-18].

Earlier studies suggested that among the PP1 isoforms, PP1δ has the greatest pRb-directed phosphatase activity [15] and co-immunoprecipitates with pRb from mitotic and early G1 cells [19]. However, detailed studies in cells at mitotic exit indicated that all three PP1 isoforms dephosphorylate pRb, and that targeting of specific sites is temporally-regulated [6]. The PP1 catalytic subunit itself is subjected to inhibitory phosphorylation by Cdks, thus preventing its premature activation and allowing coordinated cell cycle progression [20-23].

The carboxyl terminal region of pRb has been found to be both necessary and sufficient for the interaction with PP1α [24]. However, mitotic PP1 also dephosphorylates Ser/Thr residues located outside the pRb carboxyl terminus [6], suggesting an interaction with additional pRb domains. The mitotic pRb-directed PP1 has been described as a high molecular weight complex, suggesting the presence of a PP1 regulatory subunit which would target PP1 to pRb and contribute to enzyme regulation [15]. Though some candidates were proposed (e.g. PP1 nuclear targeting subunits, [17]), the presence and precise identification of such a regulatory subunit may be argued.

In the present work, we investigated the association between pRb and the PP1 isoforms, as well as the presence of PP1 regulatory subunits associated with the pRb-directed PP1. We report that the entire pRb molecule is required for optimal in vitro interaction with the PP1 isoforms derived from cell extract and that this interaction is a direct one.

## Results

### Association of PP1 from mitotic HeLa cell extracts with recombinant pRb [Fig 1]

**Figure 1:**
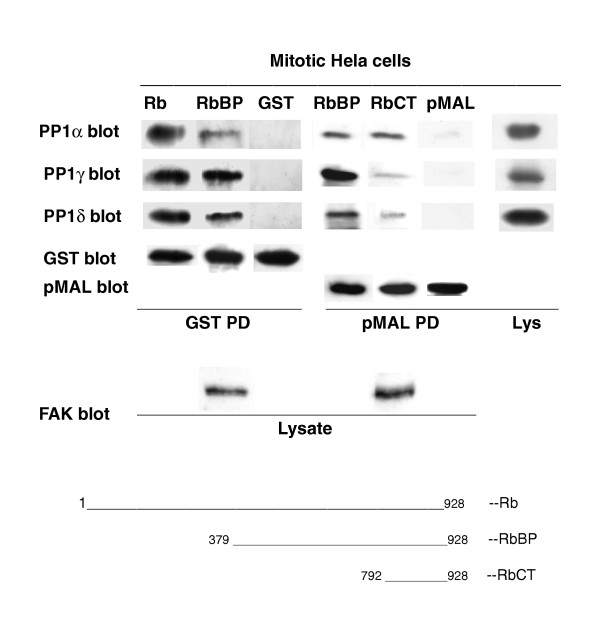
**Association of mitotic PP1 isoforms with Rb or Rb-deletion mutants**. The GST-Rb fusion proteins, full-length (Rb) and big pocket (RbBP), or the pMAL-Rb fusion proteins, RbBP and carboxyl terminus (RbCT; also indicated in the lower part of the figure), were bound to the appropriated beads and used to co-precipitate (GST PD or pMAL PD) the PP1 isoforms from mitotic Hela cells lysate (2.5 mg/PD). This was followed by electrophoresis, to which cell lysate was also added (Lys), and immunoblotting to detect the PP1 isoforms (PP1α, γ1 and δ blot) and the GST- or pMAL-fusion proteins (GST blot or pMAL blot). Detection of the unrelated FAK protein (FAK blot) in the cell lysates used for the GST or pMAL PD assays confirmed the use of equal protein amounts. The data presented are representative of multiple experiments.

**Figure 2:**
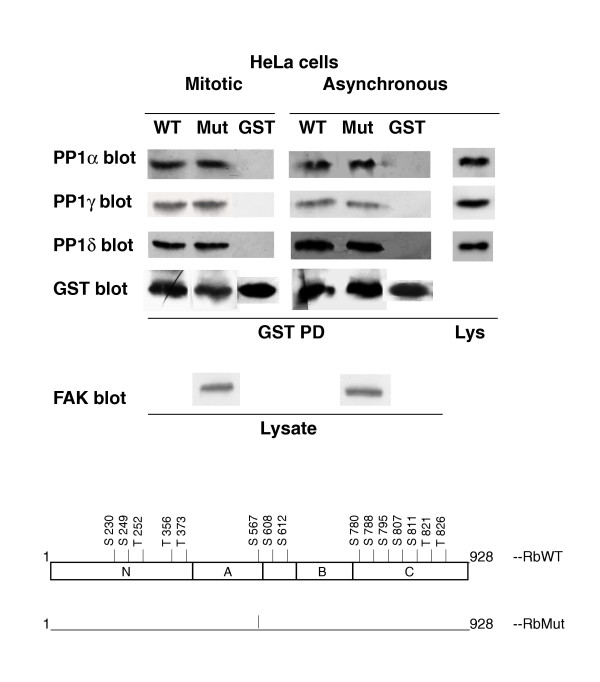
**Association of cellular PP1 isoforms with wild-type or Ser/Thr-to-Ala Rb-mutant**. GST-Rb, either wild-type (WT) or carrying the Ser/Thr-to-Ala mutations (Mut) of the sites indicated in the lower part of the figure (further described in [31]) or GST, were bound to glutathione-Sepharose beads and used to co-precipitate the PP1 isoforms from 2.5 mg of mitotic or asynchronous Hela cells extracts. All the rest was as in Fig. 1. FAK was used to normalize the amount of mitotic and asynchronous cell extracts used, since FAK protein did not change throughout the cell cycle. The data presented are representative of two independent experiments.

The carboxyl terminal region of pRb (residues 792–928) has been reported to be both necessary and sufficient for in vitro complex formation with PP1α [24]. However, the PP1 isoforms also dephosphorylate sites located in the A/B big pocket and N-terminal regions of pRb [6], (depicted in the lower part of Fig 2). To investigate whether the entire pRb molecule was required for binding the PP1 isoforms, we compared full-length pRb (GST-Rb) and pRb deletion mutants in their ability to co-precipitate PP1α, γ and δ from equal amounts of mitotic HeLa cells extracts. The mutants employed were big pocket (RbBP, residues 379–928, prepared as GST-RbBP or pMAL-RbBP) and C-terminus (RbCT, residues 792–928, prepared as pMAL-RbCT). We found that the entire pRb molecule was required for maximal association of each PP1 isoforms (detected with our isoform-specific antibodies [5,25], whereas GST or pMAL alone did not bind PP1 (Fig. 1). We also confirmed the previous data [24] that neither Rb-small pocket (GST-Rb-SP, residues 379–793) nor Rb-N-terminal (GST-RbNT, residues 1–380) were able to bind PP1 (not shown).

### Association of PP1 from mitotic or asynchronous HeLa cell extracts with pRb or Ser/Thr pRb-mutants [Fig 2]

Since PP1 interacts with pRb throughout the cell cycle [18,22], we compared the binding of all three PP1 isoforms from mitotic and asynchronous cell extracts. The results indicated that their association with pRb was quite similar (compare WT data in Fig. 2).

All the above experiments used bacterial proteins, indicating that the targeted sites may attract PP1 even in their unphosphorylated form. This is similar to what we observed with the interaction of PP1 and the Ron receptor [26] or FAK [27]. We next addressed whether the Rb phosphorylation sites were involved in the association of PP1 with pRb. To test this hypothesis, we analyzed the ability of the pRb carrying Ser/Thr-to-Ala mutations of 14 residues (indicated in the bottom part of Fig 2) to co-precipitate PP1. Surprisingly, we found that mutation of all 14 sites did not affect the ability of pRb to co-precipitate the PP1 isoforms from mitotic or asynchronous HeLa cells extracts (Fig. 2). These results support the notion that the association of PP1 with pRb is not dependent upon the presence of these Ser/Thr residues.

### Isolation of the pRb-directed PP1 holoenzyme obtained from a cell extract [Fig 3]

**Figure 3:**
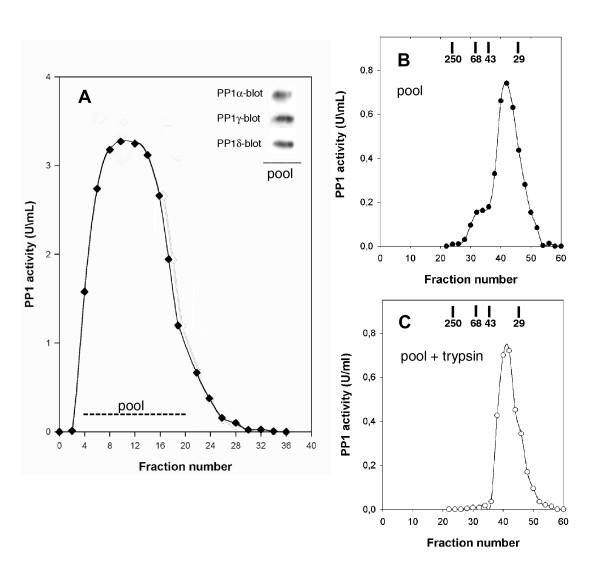
**A – Affinity chromatography of mitotic PP1 on GST-Rb-Sepharose column**. Mitotic Hela cells extract (40 mg) was applied to a 1 ml GST-Rb-Sepharose column (detailed in the Methods section). The collected fractions were assayed for PP1 activity and pooled as indicated. One aliquot of the pool was concentrated by precipitation in the presence of 7% TCA and subjected to electrophoresis and immunoblotting to detect PP1 isoforms (inset). – **B – Gel-filtration of the PP1 eluted from the affinity column**. Following concentration, one half of the activity pool was applied to an FPLC Superose 12 HR 10/30 gel filtration column. 0.2 ml fractions were collected, after discarding the first 6 ml, and assayed for PP1 activity. – **C – Gel-filtration of the trypsin-treated PP1 pool**. The remaining half of the concentrated activity pool was subjected to limited tryptic-proteolysis prior to gel filtration. Molecular weight markers: catalase (250 k), BSA (68 k), ovalbumin (43 k), carbonic anhydrase (29 k). The data presented are representative of several experiments (see Results).

We next sought to determine whether the interaction between PP1 and pRb involved a PP1-trageting subunit [1]. For this purpose we used a full-length GST-Rb affinity column to capture pRb-directed PP1 complexes from mitotic HeLa cells extract, which were subsequently analyzed by gel filtration. PP1 eluted from the affinity column as a single activity peak (Fig. 3A). Immunoblotting of the pooled peak fractions indicated the presence of all three PP1 isoforms (Fig. 3A, inset). Following concentration, the pool was analyzed by gel filtration on FPLC Superose 12 HR 10/30. The PP1 activity peak displayed an approximate 35 000 Mr, supporting our conclusion that PP1 was present as a free catalytic subunit (Fig. 3B; the experiment shown is representative of three independent experiments). Limited tryptic-digestion of the affinity column pool, performed prior to gel filtration, did not change the elution profile significantly (Fig. 3C), thus further confirming the absence of additional PP1 associated proteins. The small peak that eluted just before the major activity peak (Fig. 3B), which was removed by trypsin-treatment (Fig. 3C), may represent a non-specific aggregate, since it was also found in other PP1 forms that had been obtained from crude cellular extracts by affinity chromatography [27]. This experiment was repeated with affinity columns prepared with GST- or pMAL-RbBP and GST- or pMAL-RbCT, using mitotic or asynchronous Hela cells extracts (data not shown). These experiments revealed a single activity peak of 35,000 Mr for PP1. Taken together, the results strongly suggest the absence of a PP1 regulatory subunit in the pRb-directed PP1 obtained from either mitotic or asynchronous HeLa cells.

### Interaction of pRb with free PP1 catalytic subunit [Fig. 4]

**Figure 4:**
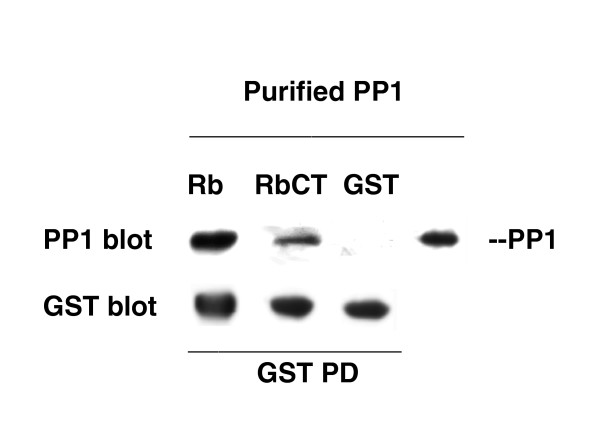
**Interaction of pRb or RbCT with PP1 catalytic subunit**. PP1 catalytic subunit (purified from muscle as a mixture of the three isoforms) was applied to a 0.5 ml column prepared with either GST-Rb-Sepharose or GST-RbCT-Sepharose or GST-Sepharose (further described in the Methods section). The PP1-containing fractions were pooled and concentrated by precipitation in the presence of 7% TCA. One aliquot of each pool was subjected to electrophoresis and immunoblotting to detect PP1 (PP1 blot, using a mixture of the three isoform-specific antibodies) and GST-Rb, GST-RbCT or GST (GST blot). PP1 catalytic subunit (30 ng loaded on the electrophoresis) is also shown as positive control. The data presented are representative of two independent experiments.

To further test the hypothesis that the interaction between pRb and PP1 is a direct one, we investigated the ability of the PP1 catalytic subunit purified from rabbit muscle (a mixture of the three isoforms) to associate with pRb or RbCT. Both full-length Rb and RbCT co-precipitated PP1 catalytic subunit (Fig. 4), supporting the conclusion of a direct association with PP1. Moreover, a greater association between pRb and PP1 was observed compared to the association between RbCT and PP1, thus confirming the results obtained with cellular-derived PP1 (Fig. 1).

### Reconstitution of a PP1-pRb complex [Fig. 5]

**Figure 5:**
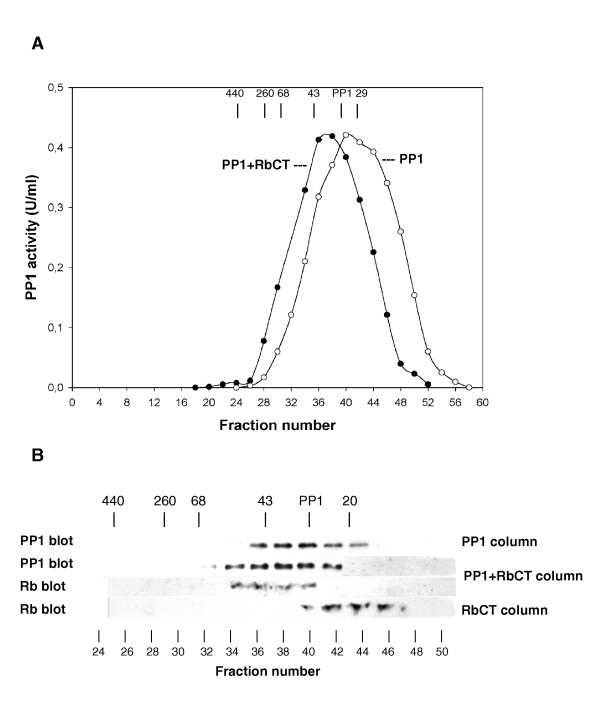
**A – Gel filtration of the PP1-RbCT complex**. PP1 catalytic subunit (as in Fig. 4) was incubated with the carboxyl terminal region of Rb (obtained from GST-RbCT by thrombin-proteolysis) in 1:1 molar ratio at 4° for 30 min and applied to a Sephacryl S400 HR gel filtration column (further described in the Methods section). The column fractions were assayed for PP1 activity (filled circles). PP1 (open circles) or RbCT were also run separately on the column (see also part B of the figure). The molecular weight markers were as in Fig 3, with the addition of ferritin (440 k) and PP1 (35 k). – **B – Immunological detection of PP1 and RbCT eluted from the gel filtration columns**. Either PP1, or the PP1-RbCT complex or RbCT were subjected to gel filtration (see part A; here indicated as PP1, PP1+RbCT and RbCT columns). The fractions obtained from the columns were concentrated by precipitation and analyzed by electrophoresis and immunoblotting to detect PP1 (PP1 blot, using a mixture of the three isoform-specific antibodies) or RbCT (Rb blot). The data presented are representative of two independent experiments.

To further confirm the direct interaction between pRb and PP1, free PP1 catalytic subunit (Mr 35 000) and RbCT (approx. Mr 14 000 after GST-removal with thrombin) were incubated in 1:1 molar ratio and subsequently analyzed by gel filtration. Gel filtration analysis of PP1 and RbCT alone were also performed. When PP1 and RbCT were present together, the PP1 activity displayed a single peak (Fig. 5A, filled circles) eluting slightly ahead of the PP1 alone peak (open circles), suggesting PP1-RbCT complex formation. This complex formation was confirmed by the immunoblotting, which detected the presence of both PP1 and RbCT in the same peak fractions (see PP1 blot and RbCT blot of the PP1+RbCT column fractions, Fig. 5B). Conversely, both PP1 and RbCT were retarded when run separately (see PP1 blot and Rb blot of the PP1 and RbCT column fractions, respectively, Fig. 5B). Taken together, the results further confirmed the ability of PP1 to interact directly with pRb.

## Discussion

In the present study we reported that optimal in vitro interaction of pRb with the three PP1 isoforms found in cellular extract requires the entire pRb molecule. This interaction was found to be direct, rather than involving a PP1-targeting subunit, and did not depend upon the presence of pRb Ser/Thr residues that are known to be PP1-targets.

A previous investigation indicated that at mitotic exit all the three PP1 isoforms dephosphorylated a number of Ser/Thr Rb sites, which were located in the C-terminal and N-terminal domains and in the region that links the A and B small pockets [6]. Additionally, the C-terminal region of pRb was reported to be both necessary and sufficient for the interaction with the PP1α isoform, whereas neither the N-terminal nor the small A/B pocket (which lacks both the N- and C-terminal domains) bound to PP1α [24]. The present results demonstrate that the C-terminal domain interacts with all three PP1 isoforms contained in cellular extract (Fig. 1). However, both full-length pRb and Rb-BP displayed greater binding capacity than RbCT with respect to all the isoforms. This is most likely due to the presence of additional PP1-binding residues or domains located outside RbCT and supports the previous report that at mitotic exit PP1 targets Ser/Thr residues located throughout the entire molecule [6].

The PP1-pRb interaction did not require phosphorylation of any site potentially targeted by PP1, since it occurred also with the non-phosphorylated recombinant pRb forms. This was not surprising since we had reported a similar result in the case of the interaction of PP1 with Ron and FAK [26,27]. More surprisingly was the finding that point-mutation of up to 14 Ser/Thr sites, most of which were demonstrated PP1 targets [6], did not affect PP1 binding. This may indicate that either the sequences surrounding the phosphorylation sites or other pRb sequences play a relevant role in PP1-binding. Indeed we arrived at a similar conclusion in the case of FAK, whereby mutation of four potential PP1-binding residues in the C-terminal domain decreased, but did not abolish, PP1-binding to FAK [27]. In the case of pRb, the presence of secondary binding-sites was suggested to explain why RbCT behaved as a non-competitive inhibitor of PP1, in spite of being a PP1 substrate [24]. Secondary binding sites were also reported in the case of another PP1 substrate, the K-Cl cotransporter [28], and might be a more general mechanism required to anchor the PP1 catalytic subunit when a targeting subunit is not available.

pRb interacts with the PP1 catalytic subunit isoforms directly. This conclusion is supported by the following evidence: 1) the PP1 from both mitotic and asynchronous cell extracts eluted as a single activity peak of approx. 35 000 Mr, representative of free catalytic subunit. The same result was obtained with the RbBP- or RbCT-directed PP1; 2) limited tryptic digestion did not change the elution profile; 3) pRb or RbBP was able to associate with purified PP1 catalytic subunit; 4) a stable complex was reconstituted from free PP1 catalytic subunit and RbCT. These properties seem to apply to all three PP1 isoforms, since they were found to co-associate with pRb in the co-precipitation experiments. In addition all three PP1 isoforms were found in the Rb-directed PP1 activity peak isolated by affinity chromatography. Taken together, our results suggest that during mitosis, all the three PP1 isoforms may associate directly with pRb, ready to perform the sequential Ser/Thr dephosphorylations occurring at M-to-G1 transition.

## Conclusion

In summary, the work supports the conclusion that full length pRb is required for optimal interaction with all the three PP1 isoforms in vitro. Surprisingly, the phosphate-accepting residues on pRb did not appear to direct this interaction. The association of all three isoforms of PP1 with pRb was a direct one, since no evidence of PP1-associated regulatory subunit was detected in the pRb-directed PP1 activity or required for complex reconstitution.

## Methods

### Antibodies, enzymes and other chemicals

PMSF, benzamidine, TPCK, leupeptin, protein A-Sepharose, protein A-peroxidase, nocodazole, media and additives for cell culture were purchased from Sigma Chem. Co.. Glutathione-Sepharose 4B, the pGEX vectors, anti-GST antibodies and thrombin were from Amersham Biosciences. Amylose resin, the pMAL-c2E vector and the restriction enzymes were from New England Biolabs. Taq DNA polymerase and the rapid DNA ligation kit were from Roche Molecular Biochemicals. The affinity-purified anti-pRb polyclonal antibody (C-15, recognizing both hyper- and hypophosphorylated pRb forms) and the anti-pMAL antibody were from Santa Cruz. The anti-GST antibody was from Amersham Biosciences. The three PP1-isoform-specific antibodies (hyperimmune serum) were raised by us in rabbits injected with C-terminal peptides [5]. PP1 catalytic (35 000 Mr) was purified from rabbit muscle [29] and did not contain PP2A.

### Plasmid preparations

Full-length GST-pRb, GST-Rb-big pocket (RbBP, residues 379–928), GST-Rb-C-terminal (RbCT, residues 792–928) were described previously [24,30]. For the pMAL-fusion proteins of RbBP and RbCT, the amplimers were produced by PCR using the following synthetic oligonucleotides (Primm, I): 5'-GAATTCATGAACACTATCCAACAA-3' and 5'-GGATCCTCATTTCTCTTCCTTGTT-3' (antisense) for RbBP; 5'-GAATTCCCTAGTTCACCCTTACGG-3' and 5'-GGATCCTCATTTCTCTTCCTTGTT-3' (antisense) for RbCT. The template DNA was either RbBP or RbCT cDNA in pGEX vector [30]. The RbBP fragment was cloned into the pGEM-T vector, cut at the *BamHI *sites and subcloned into the same site of the pMAL-c2E vector. The RbCT fragment was cloned into the pGEM-T vector, cut at the *BamHI *and *EcoRI *sites and subcloned into the same sites of the pMAL-c2E vector. E. coli BL-21 protease-minus bacteria were used for the pMAL- or GST-fusion protein production. The phosphorylation sites mutant of full-length GST-Rb (produced in *E. coli *Bl21pLys) was described previously [31].

### Expression of fusion proteins in bacteria

For GST-fusion proteins production, the bacteria were grown at 37°C in LB-Ampicyllin (or 2XYT-Ampicyllin supplemented with 0.1% w/v glucose and 20 mg/ml chloramphenicol in the case of E. coli Bl21pLys), induced with 0.1 isopropyl-β-D-thiogalactoside (IPTG) at 37°C for 2 h or 0.2 mM IPTG at 20°C for 16 h, collected by centrifugation and resuspended in 25 mM TRIS-HCl, pH 7.5, 150 mM NaCl, 15 mM 2-mercaptoethanol (bacterial lysis buffer) added with protease inhibitors (0.02 % w/v benzamidine, 0.02 % w/v PMSF, 0.02 % w/v TPCK and 4 μg/ml leupeptine). Following quick freezing and thawing, 0.25 % v/v Triton X-100, 50 U/ml DNase, 10 mM MnCl_2 _and 10 mM MgCl_2 _were added, followed by 30 min rotation at 4°C and 10,000 × *g *centrifugation for 20 min. The extract thus obtained was either used immediately in pull-down assays or stored at -20°C.

### Cell growth and extracts

Hela cells were grown at 37°C in water-saturated CO_2_, in DMEM added with 10 % v/v fetal calf serum (Sigma Chem. Co.). Following two washes in cold PBS, cells were lysed in 50 mM TRIS-HCl, pH 7.5, 250 mM NaCl, 5 mM EDTA, 0.1 % Triton X-100 (cell lysis buffer), 7.5 mM 2-mercaptoethanol, 1 mM orthovanadate and protease inhibitors as described for bacterial extracts. Sub-confluent Hela cells were exposed to 50 ng/ml nocodazole for 18 h. The mitotic cells (approximately 50% of the cells) were collected by gentle pipetting, washed in cold PBS and lysed.

### Co-precipitation assays and Western blots

GST-proteins from bacterial extracts were bound to 50 μl of glutathione-Sepharose beads pre-treated with lipid-free BSA. pMAL-fusion proteins from bacterial extracts were bound to 50 μl of Amylose resin, according to the manufacturer's instructions. After 90 min rotation at 4°C and three washes with bacterial lysis buffer (added with 0.1 %Triton X-100, 0.02 % w/v benzamidine and 0.02 % w/v PMSF), the beads were mixed with 2–3 mg of cell extract. This was followed by incubation at 4°C for 90 min with shaking, three washes in cell lysis buffer added with 0.02 % w/v benzamidine and 0.02 % w/v PMSF and boiling in Laemmli buffer. Electrophoresis was on 8.5 % polyacrylamide-SDS gel and Immobilon-P membranes (Millipore) were used for transblotting. Immunoblotting was carried out with the indicated antibodies, followed by anti rabbit-peroxidase (Sigma Chem. Co.) and the enhanced chemiluminescence ECL system (Amersham Biosciences).

### Affinity chromatography and gel filtration

GST- or pMAL-fusion proteins (25 or 50 ml of bacterial extract) were bound to 1 or 2 ml of Glutathione-Sepharose beads, according to the manufacturer's instructions. The resin was washed 4 times in bacterial lysis buffer, mixed with 30–40 mg of cell extract, rotated for 90 min at 4°C, washed extensively with cell lysis buffer containing 25 mM TRIS-HCl, pH 7.5, 150 mM NaCl, 15 mM 2-mercaptoethanol, 0.02 % w/v benzamidine and 0.02 % w/v PMSF and transferred to a 2 ml Bio-Rad Poly-prep column. The column was run at 5.5 ml/h and directly eluted with cell lysis buffer containing 300 mM NaCll. 0.640 ml fractions were collected and assayed for PP1 activity with the substrate [^32^P]Phosphorylase *a *[19,23]. 1 unit of PP1 releases 1 nmol of [^32^P]H_3_PO_4_/min at 30°C and is calculated after subtracting the cpm of blanks without enzyme. For immunoblotting to detect the PP1 isoforms, the peak fractions were pooled, concentrated by precipitation in the presence of 7 % TCA and boiled in Laemmli buffer. For gel filtration, the peak fractions were pooled, concentrated using Amicon Centricon (Millipore) and loaded on an FPLC Superose 12 HR 10/30 gel filtration column (Amersham Biosciences), equilibrated in 10 mM imidazole, pH 7.5, 5 % glycerol, 0.01 % Brij-35, 100 mM NaCl, 15 mM 2-mercaptoethanol, 0.02 % w/v benzamidine and 0.02 % w/v PMSF. The column was run at 24 ml/h. 0.20 ml fractions were collected after discarding the 6 ml void volume, and assayed for PP1 activity. An aliquot of the concentrated pool was treated with trypsin (20 μg/ml) for 15 min at 30°C, followed by excess soybean trypsin inhibitor, and analyzed by gel filtration.

### Association between PP1 and Rb-CT

GST-RbCT fusion protein (96 ml of bacterial extract) were coupled to 1 ml of glutathione-Sepharose beads, according to the manufacturer's instructions. The resin was washed 3 times with bacterial lysis buffer and once with thrombin cleavage buffer containing 50 mM TRIS/HCl, pH 8.0, 150 mM NaCl and 2.5 mM CaCl_2_. After centrifugation, the beads were resuspended in 1 ml of thrombin cleavage buffer containing 48 U of thrombin, shaken at room temperature for 40 min and centrifuged to collect the supernatant containing free RbCT (Mr 14 000), which was then concentrated using Amicon Centricon. PP1 catalytic subunit (Mr 35 000), purified from rabbit muscle as a mixture of the three isoforms [29], was incubated with RbCT (1:1 molar ratio) at 4°C for 30 min and then loaded on a 0.8 × 48 cm Sephacryl S-400 HR gel filtration column (Amersham Biosciences), equilibrated with 25 mM imidazole, pH 7.5, 100 mM NaCl, 1 mM EDTA, 0.1% TX-100, 15 mM mercaptoethanol, 0.02% w/v benzamidine and 0.02% w/v PMSF. The column was run at 10.5 ml/h and 0.26 ml fractions were collected after discarding the void volume. The fractions were assayed for PP1 activity and the activity peak fractions were pooled and concentrated by precipitation in the presence of 7% TCA, followed by electrophoresis and immunoblotting, to detect PP1 or RbCT.

## Abbreviations

PP1, type-1 protein Ser/Thr phosphatase; pRb, full-length retinoblastoma gene product; RbBP, Rb-big pocket, including A/B pocket and the C-terminal domain; RbCT, Rb C-terminal domain; Cdks, cyclin-dependent kinases; LB-broth, Luria-Bertani broth; IPTG, isopropyl b-D-thiogalactopyranoside; GST, glutathione-S-tranferase; DMEM, Dulbecco's modified Eagle's medium; PBS, phosphate-buffered saline; TRIS, Tris-(hydroxymethyl)aminomethane); EDTA, Ethylenediaminetetracetic acid; TPCK, L-1-p-tosylamino-2-phenylethyl chloromethyl ketone; PMSF, phenylmethyl sulfonyl fluoride; BSA, bovine serum albumin; SDS, sodium dodecyl sulfate; TCA, trichloroacetic acid.

## Competing interests

The author(s) declare that they have no competing interests.

## Authors' contributions

MV carried out most of the experiments and contributed to manuscript drafting; MB contributed to cell culture and western blot experiments; JWL and SM supplied DNA constructs, participated in experimental design and discussion, as well as in critical manuscript revision. EVM envisaged the study, participated in its design and coordination as well as in manuscript drafting. All authors approved the final manuscript.
